# Associations between Public Fear of COVID-19 and Number of COVID-19 Vaccinations: A County-Level Longitudinal Analysis

**DOI:** 10.3390/vaccines10091422

**Published:** 2022-08-29

**Authors:** Jingjing Gao, Yuqi Guo, Lilian Ademu

**Affiliations:** 1 Texas A&M AgriLife Center in El Paso, Texas A&M University, El Paso, TX 79927, USA; 2 School of Social Work, College of Health and Human Services, University of North Carolina at Charlotte, Charlotte, NC 28262, USA; 3 School of Data Science, University of North Carolina at Charlotte, Charlotte, NC 28262, USA; 4 Public Policy Program, College of Arts and Sciences, University of North Carolina at Charlotte, Charlotte, NC 28262, USA

**Keywords:** COVID-19, vaccination, lottery, bonus

## Abstract

Background and Purpose: A large number of COVID-19 infections and deaths and the ensuing socioeconomic problems created widespread public fear around COVID-19. Fear around COVID-19 greatly influences people’s attitudes towards receiving the COVID-19 vaccines. The purpose of this study is examining (a) the impact of the public fear of COVID-19 (PFC) on the number of COVID-19 vaccinations at the county level; (b) the interaction effect between the PFC and per capita income, unemployment rates, and COVID-19 vaccines incentive policies, on the number of COVID-19 vaccinations at the county level. Method: This is a longitudinal analysis across states in the U.S. by using county-level data of 2856 counties from 1 February to 1 July. Random-effects models were adopted to analyze the associations between the PFC and the number of COVID-19 vaccinations. Result: the PFC was positively associated with the number of COVID-19 vaccinations at county-level, as PFC increases from 0 to 300, the predicted vaccination number increases from 10,000 to 230,000. However, the associations were divergent when the PFC interacts with county-level per capita income, unemployment rates, and incentive policies. Conclusion: public fear is an important indicator for the county-level vaccination numbers of COVID-19. However, it is critical to consider public fear and socioeconomic factors when making policies that aim to increase COVID-19 vaccination rates.

## 1. Introduction

Up to June 2022, severe acute respiratory syndrome coronavirus 2 (COVID-19) has caused 83,949,036 infected cases and 1,002,067 deaths in the United States (U.S.) [1]. A large number of COVID-19 infections and deaths and the ensuing socioeconomic problems (e.g., social alienation, travel ban, high unemployment, and inflation) created widespread public fear around COVID-19 [2,3,4,5,6,7,8,9,10,11]. This unprecedented public health crisis stresses the whole society with mental disturbance [12,13]. Fear is one of the primary emotions that people feel when faced with a threatening situation [14,15]. A public emotion like fear is a critical stimuli in a public health crisis [13]. A variety of uncertainties of COVID-19 threatens people’s health and lifestyle. The absence of effective COVID-19 treatment and socioeconomic damage inevitably leads to public fear.

Public emotion is an important indicator of health behaviors in public health management. Fear affects people’s health-related risk perceptions and decision-making processes [16,17,18,19]. A robust body of research has documented that fear of diseases will increase the public’s preventive health care behaviors, such as smoking, vaccination, and alcohol use [20,21,22,23,24]. Under circumstances with high uncertainties, emotions have a more significant role in decision making because people tend to make a health care decision based on feelings and cognitive shortcuts when not much reliable information is available (WHO, 2017).

COVID-19 vaccines can effectively prevent severe disease, hospitalization, and death from COVID-19 [25,26]; thence, vaccination is a major strategy to save lives from COVID-19 and combat the COVID-19 crisis in the U.S. Fear of the new COVID-19 virus and associated socioeconomic issues undoubtedly influence people’s action toward receiving the COVID-19 vaccines [27,28]. Understanding how public fear emotion during a public health crisis impacts populations’ preventive vaccine use is critical to the future success of public health management.

Public emotion is highly influenced by public health policies and socioeconomic factors during a public health crisis. The COVID-19 pandemic is particularly stressful for people with no work, and low income, as they are more financially burdened with physical activity limits during the pandemic [29,30]. As unemployment is one of the most significant social problems accompanied by COVID-19. Lost job has been documented as a significant factor that leads to fear and anxiety [31]. Income and unemployment, meanwhile, are significantly associated with the COVID-19 vaccine uptake [32,33]. A national analysis found that counties with higher per capita income and unemployment rates had higher rates of COVID-19 vaccination [32]. Additionally, the effectiveness of policy implementation and public emotion are mutually reinforcing [34,35,36]. Lottery incentive policies and bonus incentive policies can effectively increase county-level COVID-19 vaccination rates in the U.S [37]. However, the role of public fear on the effectiveness of vaccine incentive policies on COVID-19 vaccinations is not clear in the current literature.

With the rapid development of social media and technologies, social network data are widely mined to gather public emotions [38,39,40,41]. Narratives of posts on social media instantly record people’s experiences and emotions during a public crisis. Fear has been identified as one of the key emotions embedded in these social media narratives when facing the COVID-19 public crisis [42]. It is well documented that fear is associated with preventative behaviors, including adherence to the lockdown rules, professional turnover intentions, wearing masks, and social distancing behavior [43,44,45,46,47]. However, how public fear impacts on COVID-19 vaccine uptake is understudied in the current literature. Our study will fill this gap by examining the relationship between public fear towards COVID-19 and the county-level number of COVID-19 vaccinations across the U.S. In this study, we will use a large amount of Twitter data to capture the public fear of the COVID-19 outbreaks. We aimed to examine (a) the impact of the public fear of COVID-19 (PFC) on the number of COVID-19 vaccinations at the county level; (b) the interaction effect between the PFC and per capita income, unemployment rates, and COVID-19 vaccines incentive policies, on the number of COVID-19 vaccinations at the county level.

Hypothesis:

**Hypothesis** **1** **(H1).** *The PFC is positively associated with the number of COVID-19 vaccinations at the county level*.

**Hypothesis** **2** **(H2).** *There is a significant interaction between the PFC and unemployment rates on the number of COVID-19 vaccinations at the county level*.

**Hypothesis** **3** **(H3).** *There is a significant interaction between the PFC and per capita income on the number of COVID-19 vaccinations at the county level*.

**Hypothesis** **4** **(H4).** *The COVID-19 vaccine incentive policies moderate the effects of the PFC on the number of COVID-19 vaccinations at the county level*.

## 2. Method

### 2.1. Research Design

This is a longitudinal analysis across states in the U.S. by using county-level data. Our panel data includes 2856 counties across 50 states from 1 February to 1 July 2021. Texas is excluded from this study because Texas’s law (Texas Health and Safety Code Sec. 161.0073) does not allow the state to release the COVID-19 vaccination data. This study uses multiple sources to analyze the relationships between the PFC and the number of COVID-19 vaccinations. The number of COVID-19 vaccinations at the county level were obtained from the U.S. Centers for Disease Control and Prevention’s (CDC) COVID-19 Vaccine Tracker [48]. The PFC was mined from the Twitter COVID-19 Data Feed, which is a real-time feed from Twitter [49,50]. The content of the stream is selected by Twitter based on parameters that contain content specific to COVID-19. Data on the COVID-19 vaccine incentives policies were provided by National Government Association (https://www.nga.org/wp-content/uploads/2021/05/Vaccine-Incentives-Memo-6.23.2021.pdf, accessed on 30 July 2021), and data on the COVID-19 vaccine distribution phases in each county were provided by the Kaiser Family Foundation (https://www.kff.org/coronavirus-covid-19/issue-brief/the-covid-19-vaccination-line-an-update-on-state-prioritization-plans/, accessed on 11 January 2021). The county-level socioeconomic characteristics were obtained from the U.S. Census [51].

### 2.2. Dependent Variable

The dependent variable, the number of COVID-19 vaccinations, is the total number of adults aged 18 or above who have been fully vaccinated, including adults with either two doses of the Pfizer/Moderna vaccines or one dose of the Johnson and Johnson vaccine. We controlled the log value of the total population per county across the U.S.

### 2.3. Independent Variables

The key independent variable is the PFC. We measured the public emotion of fear toward COVID-19 during the outbreak of this pandemic by detecting the perception of COVID-19 risk in people’s social media posts on Twitter. This study used Test2emotion (https://towardsdatascience.com/text2emotion-python-package-to-detect-emotions-from-textual-data-b2e7b7ce1153, accessed on 14 September 2020). This package is developed by Aman Gupta, Amey Band, Shivam Sharma, Karan Bilakhiya, a Python package, which was designed to identify public emotions through tweets of Twitter data [52]. Texa2emotion detects emotions embedded in any textual data and categorizes these emotions into five categories–Happy, Angry, Sad, Surprise, and Fear. Research has increasingly used Test2emotion in the public health field to detect public emotions, including COVID-19 studies [53,54,55]. We first used Test2emotion to mine the PFC from each tweet. Then the levels of fear in each tweet were evaluated and assigned a number (0–1) by the Test2emotion to calculate the fear index. We then aggregated the fear index from individual levels to county levels based on each tweet’s geographical location information. There are 9330 observations with PFC values of zero, because most of these counties are in rural areas. The population size in these counties is relatively small, and people in these counties are less likely to use social media because of the digital divide [56,57,58]. Moreover, 95% of PFC values collected in this study are 52.29 or lower, so we use 52.29 of PFC as the cutline for a high level of PFC and zero to represent a low level of PFC.

In this study, we controlled COVID-19 vaccination-related policies, COVID-19 case numbers, health care resources, per capita income, unemployment rate, political ideology, education level, race, and age. COVID-19 vaccine incentive policies were categorized into three categories: 0 = no incentive policy, 1 = bonus incentives, and 2 = lottery incentives. Counties were coded as having bonus incentives if their state government only provided bonuses such as food, tickets to entertainment facilities, or a small amount of cash to motivate communities to take COVID-19 vaccines. Counties were coded as having lottery incentives if their state government issued a large amount of cash lottery for people who have received COVID-19 vaccines within a contained period. Additionally, we controlled the initial vaccine distribution strategies. Counties were coded into two categories: (1) counties in states that followed the vaccine coverage distribution plan proposed by the Advisory Committee on Immunization Practices (AICP) in phase 1a of COVID-19 vaccine distribution, which mainly covers healthcare workers; and (2) counties in states that that expanded the vaccine coverage to more groups such as by including people aged 65 and above in phase 1a [59].

Per capita income and the unemployment rate were measured at the county level. The local political environment was measured by the rate of people voting for Biden at the county level in the 2020 presidential election. Education level was measured by the percentage of adults with bachelor’s degrees and/or graduate degrees at the county level. Race was measured by the rate of Black, Indigenous, and People of Color populations (BIPOC) by county. Age was measured by the rate of the population aged 65 and above per county. Health care resource was measured by each county’s total nurse practitioners and the data are from the U.S. Bureau of Labor Statistics.

### 2.4. Statistical Analysis

Measures of central tendency and frequency distribution were used to describe the characteristics of the study sample. Random-effects models were adopted to examine the relationships between the PFC and numbers of the COVID-19 vaccine uptake at the county level, given other covariates of the socioeconomic characteristics of counties and COVID-19 vaccine incentive policies. Random-effects model is a kind of hierarchical linear model and we do not adopt a stepwise approach for our analysis. Four random-effects models were developed to analyze (1) the impact of the PFC on numbers of the COVID-19 vaccinations at the county level; (2) the interaction effect between the PFC and per capita income on the numbers of the COVID-19 vaccinations at the county level; (3) the interaction effect between the PFC and unemployment rates on numbers of the COVID-19 vaccinations in county-level; (4) the moderate effect of COVID-19 vaccine incentive policies on the relationship between e PFC on numbers of the COVID-19 vaccinations in the county level.

## 3. Results

**Figure A1:**
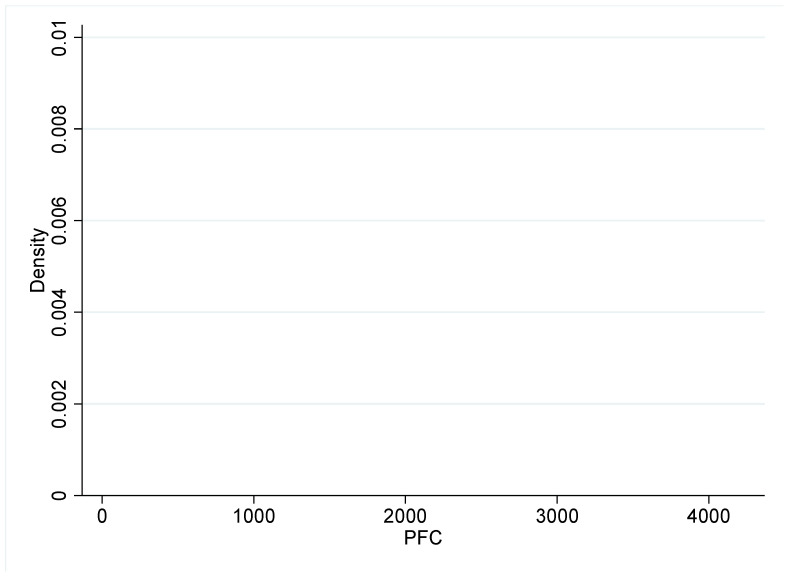
Distribution of PFC Values.

**Figure A2:**
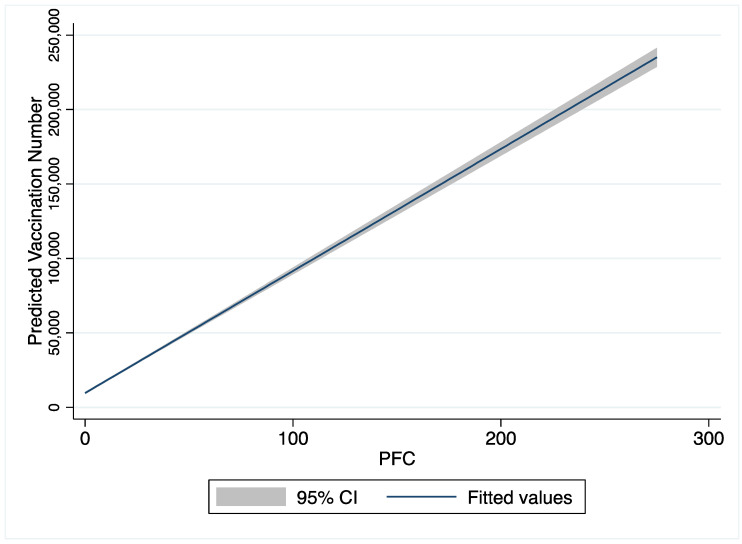
Trend of Predicted Vaccination Number by PFC Values.

Table 1 provides the descriptive statistics across 16,976 county-time-waves (2856 counties from 1 February to 1 July 2021). At the county level, the average COVID-19 vaccinated number was 23,026.71 (SD = 98,075.98); and the average number of daily new COVID-19 cases was 710.89 (SD = 3868.21). The mean PFC value was 23.06 (SD = 132.91), and Figure A1 in Appendix A shows the distribution of PFC values. Figure A1 shows that the PFC values are not normally distributed. The Skewness value of PFC (17.02) is less than the mean value of PFC (23.06), which confirms that PFC values are skewed to the left. The Kurtosis value of PFC (437.02) confirms that PFC values are heavy-tailed distribution. The distribution of PFC is not normally distributed, and it corresponds to the reality that most of the counties in the U.S are in rural areas and population density in these counties is relatively low, and people in these counties are also less likely to use social media [56,57,58]. To alleviate these two issues, we control the population size in our model. Figure A2 in Appendix A shows the overall trend of vaccination number by PFC values–as PFC increases from 0 to 300, the predicted vaccination number increases from 10,000 to 230,000. Regarding the vaccine policies: the average number of days the vaccine is available to the public in each county was 29.07 (SD = 34.04), and the average number of days to implement the vaccine incentives per county (including bonus and lottery) was 2.86 (SD = 9.54). The average per capita income at the county level was $25,091.53. Averagely, 21.83% of adults had a bachelor’s degree, 15.47% of adults were BIPOC populations, and 19.41% of adults were 65 years old or older across counties. 

As our study is longitudinal, random-effects models are preferred to explain the interaction effects because it accounts for the time component. Table 2 presents the analysis results of random-effects models. The public fear of COVID-19 (PFC) was positively (*p* < 0.05) associated with the number of COVID-19 vaccinations. For one unit increase in the fear index, the county-level COVID-19 vaccination number increased by 109. Additionally, lottery policies were positively (*p* < 0.05) associated with the number of COVID-19 vaccinations. Compared with counties without any incentive policies, counties with lottery policies had a total 2935 increase in the number of COVID-19 vaccinations.

**Figure 1:**
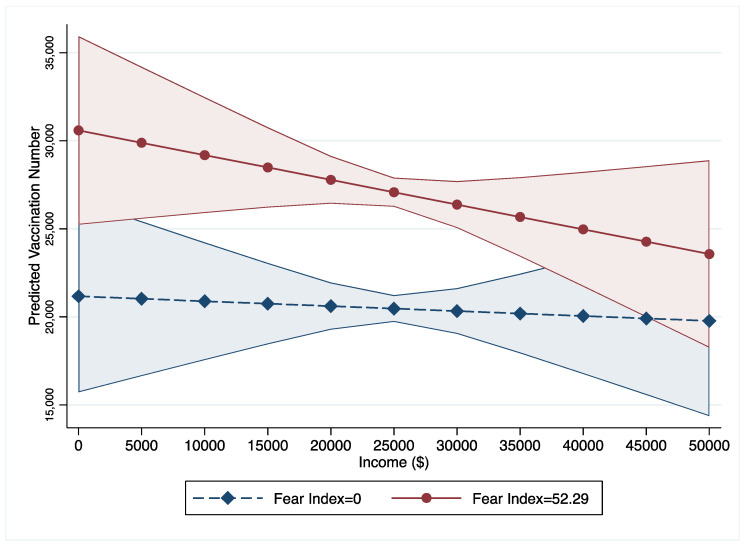
Interaction Effects between PFC and Income. Note: PFC value of 52.29 is the 95% interval and represents county-level communities with a high fear level.

**Figure 2:**
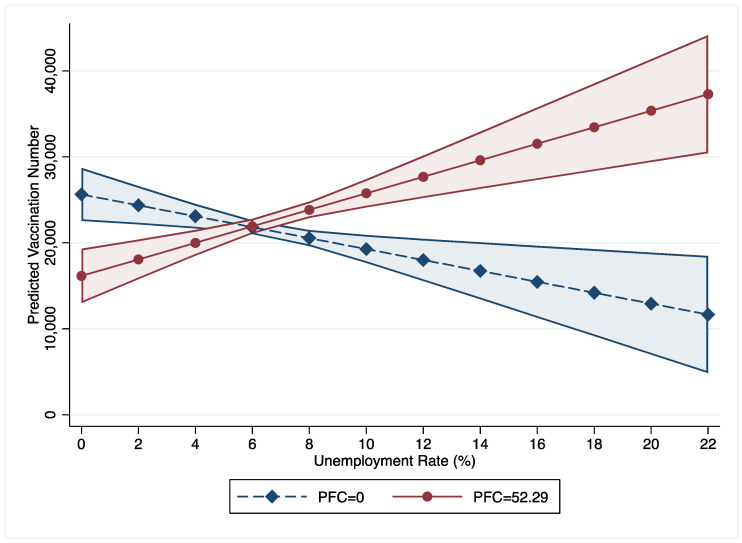
Interaction Effects between PFC and Unemployment Rate.

**Figure 3:**
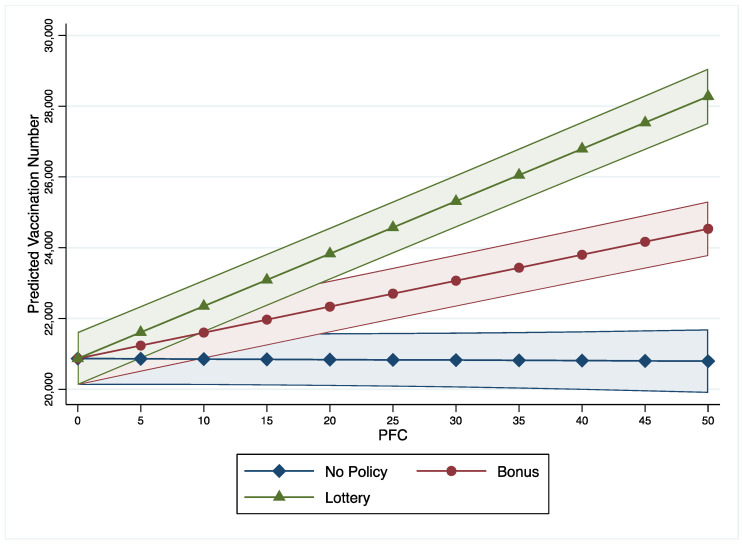
Interaction Effects between PFC and Vaccine Policies.

Significant interaction effects are found between per capita income, unemployment rates, incentive policies, and the PFC on COVID-19 vaccine uptake (models 2 to 4). As 95% of counties have a PFC value below 52.29, this study treats PFC with 52.29 as a high or strong level of fear while treating PFC with zero as a low level of fear. This study uses these two levels to show the interaction effects between income, unemployment rate, incentive policies, and PFC. First, as income increases, the vaccination numbers in counties with low PFC are lower than in counties with high PFC (Figure 1). Furthermore, the gaps between counties with low and high PFC were statistically significant when the income fell in the range between $5000 and $37,000. Figure 2 illustrates that number of COVID-19 vaccinations decrease along with the increase of unemployment rate in counties with low PFC (=0) while vaccination numbers increase in counties with high PFC (=52.29). Figure 3 shows that as the PFC increases, the number of COVID-19 vaccinations have different trends in counties with different incentive policies: (1) as the PFC increases, the number of COVID-19 vaccinations in counties with bonus policies marginally decreases from 20,000 to 10,000; (2) as the PFC increases, the numbers of COVID-19 vaccinations in counties with lottery policies significantly increases from 20,000 to 35,000; (3) as the PFC increases, the numbers of COVID-19 vaccinations in counties without incentives do not show a significant change.

Additionally, the random-effects model 1 shows that the number of nurse practitioners (*p* < 0.05) is positively associated with the number of COVID-19 vaccinations. However, the percentage of the BIPOC population (*p* < 0.05), the number of daily new COVID-19 cases (*p* < 0.05), and the percentage of adults with bachelor’s degrees (*p* < 0.05) are negatively associated with the number of COVID-19 vaccinations.

## 4. Discussion

This study analyzed the relationship between the PFC and the county-level number of COVID-19 vaccinations, as well as the interaction effects between the PFC and county-level socioeconomic factors (i.e., per capita income, unemployment rate, and vaccination incentive policies) on the number of COVID-19 vaccinations. The findings partially supported our hypotheses. First, this study found that public fear of the COVID-19 pandemic is positively associated with the number of vaccinated adults for COVID-19 at county levels. This finding is consistent with the previous studies that public fear and anxiety, can promote public preventive health care use [60,61,62,63,64,65]. Our study adds extra evidence to the current literature that public fear is a significant predictor of COVID-19 vaccine use during a public health crisis. Fear levels of COVID-19 potentially reflect communities’ concerns and their vulnerability which is associated with their subsequent prevention behaviors–vaccine uptake. Counties with higher PFC were more eager to use COVID-19 vaccines to alleviate the stressful social and economic situation caused by COVID-19 [66]. 

Second, this study found that counties with relative intense PFC still have a higher umber of COVID-19 vaccinations than counties with low or mild PFC at the per capita income range from $5000 to $36,000. However, this trend of the positive association between vaccinations and the PFC tends to moderate as county-level per capita income increases. Counties with per capita incomes between $5000 and $36,000 are in a vulnerable financial position, so people may be more likely to receive COVID-19 vaccines to contain the crisis and mitigate the economic damage caused by COVID-19. People in counties with high per capita income (>$36,000) may have more affluent health resources and the other options (e.g., working from home) to avoid exposure to COVID-19, rather than relying solely on COVID-19 vaccines, which can moderate the role of PFC on increasing the number of COVID-19 vaccinations. Given the potential risks of COVID-19 vaccines and conspiracy theories associated with the COVID-19 vaccines, people with high income may prefer to delay in receiving the COVID-19 vaccines [32].

Third, we found that this trend (increases in the PFC being associated with increases in the number of COVID-19 vaccinations) was divergent in the context of interactive effects with counties’ unemployment rates. The trend of association between the PFC and the number of COVID-19 vaccinations changes at the point of 6% of the county-level unemployment rate. Below 6% unemployment rates, the number of COVID-19 vaccinations are higher in counties with mild PFC than counties with intense PFC, but this tend reverses when counties’ unemployment rates are above 6%. These findings are supported by economics and political science. Economists generally consider 4–5% unemployment rates natural and should not be a policy concern [67,68]. Our study identified the 6% unemployment rate as the cross point of the impact of the PFC on the number of COVID-19 vaccinations, which is close to the natural unemployment rate. Fear of COVID-19 is not an effective driver of having COVID-19 vaccines in counties with a natural unemployment rate. However, fear of COVID-19 motivates people to receive COVID-19 vaccines in counties with high unemployment rates to avoid losing a job or find a new job.

Last, our study found that PFC moderates the effectiveness of incentive policies on the COVID-19 vaccination. With the PFC increasing, the number of COVID-19 vaccinations decrease in counties with bonus incentive policies, but the number of COVID-19 vaccinations increase in counties with lottery incentive policies. This finding has an important political implication. Policymakers should adopt lottery incentives to promote vaccination rates if they detect a high-level public fear during a public health crisis. The positive relationship between PFC and preventative health behavior (vaccination) found in this study is consistent with studies from other countries, including Nigeria, India, and Turkey [66,69,70,71]. However, because of policy, society, and cultural differences, more studies are needed to evaluate the interaction effects between PFC and policies and culture on preventative health behaviors.

With these notable findings above, this study has some limitations. Although social media data is an effective strategy to measure public emotion, using social media data from Twitter may have potentially excluded people who do not use Twitter. In some rural areas, where fewer people use social media, they are less likely to provide information about their geographic location. Their PFC information may be missed by Test2emotion, so the observed PFC value may be 0. Furthermore, public fear in tweets that do not have information of geographical location could not be captured in this study. In addition, counties in Texas were not included in this study because the Texas state government did not release Texas’s county-level data. Therefore, the findings of this study cannot be applied to Texas. Last, causality between the PFC and total county-level vaccination number cannot be confirmed by this study given the research design and statistical method.

## 5. Conclusions

Our study found that the PFC is positively associated with the county-level number of COVID-19 vaccinations. However, the role of the PFC on the number of COVID-19 vaccinations is influenced by socioeconomic factors (per capita income and unemployment rate) and COVID-19 inventive policies (lottery policies and bonus policies). Taken together, it is critical to consider public fear and socioeconomic factors when making policies that aim at increasing COVID-19 vaccination rates. Detecting public emotion through social media to develop public health measures is a promising strategy for future policy-making efforts. We encourage future research to continue exploring other public emotions’ (e.g., anger, anxiety, and sadness) impact on COVID-19 vaccine uptake.

## Figures and Tables

**Table 1:** Descriptive Statistics.

Variable	Mean	Std. Dev.	Min	Max	Median	Interquartile Range (IQR)	N
Vaccine uptake	23,026.71	98,075.98	0	4,804,352	3505.50	10,883	17,142
Vaccine uptake rate by population	0.19	0.15	0	2.27	0.17	0.24	17,136
PFC	23.06	132.91	0	4286.00	22.90	4.00	17,142
New infected	710.89	3868.21	0	33,0525	711.08	362.00	16,982
Days of vaccines availability	29.07	34.04	0	107	29.25	57.00	17,142
Days of incentive policy	2.86	9.54	0	55	2.87	0.00	17,142
Biden support proportion	34.07	15.86	4.97	92.15	0.34	0.21	17,142
Total nurse practitioners	54.58	156.17	0.23	3937.77	54.29	31.85	17,142
Unemployment rate 2020	6.72	2.23	1.7	22.5	6.71	2.80	17,142
Per capita income ($)	25,091.53	5996.03	9688.43	66,518.36	25,074.69	6957.57	17,142
Percent of bachelor’s degree	21.83	9.55	5.40	78.50	21.82	11.00	17,142
Percentage of Black and Indigenous People of Color (BIPOC)	15.47	16.11	9.11	93.71	0.16	0.17	17,136
Percentage of population aged 65 and above	19.41	4.56	4.83	57.58	0.19	0.05	17,136
Population log	10.33	1.46	6.14	16.13	10.32	1.79	17,136

Note: the number observation is 19,832 with 2856 counties from six-time points, which is the first day of each month from February to July.

**Table 2:** Time series analysis of public emotion and vaccination.

	(1)	(2)	(3)	(4)
		Interaction Models		
Variables	Initial Model	Income	Unemployment	Vaccine Policy
PFC	109.1 ***	180.1 ***	−181.5 ***	4.917
	(4.208)	(13.41)	(11.67)	(6.787)
New infected	−11.52 ***	−11.57 ***	−11.82 ***	−11.58 ***
	(0.124)	(0.124)	(0.122)	(0.122)
Days of vaccines availability	245.2 ***	244.4 ***	242.9 ***	247.2 ***
	(12.78)	(12.77)	(12.52)	(12.60)
Days of incentive policy	217.8 ***	217.6 ***	214.0 ***	200.9 ***
	(48.19)	(48.15)	(47.22)	(47.51)
Biden support proportion	6206	5146	3416	4470
	(4470)	(4470)	(4381)	(4411)
Total nurse practitioners	614.0 ***	612.1 ***	579.9 ***	601.4 ***
	(4.678)	(4.686)	(4.759)	(4.667)
Unemployment rate	−114.3	−117.1	−635.8 **	−69.31
	(230.4)	(230.2)	(226.6)	(227.4)
Per capita income ($)	−0.148	−0.0280	0.0795	−0.130
	(0.108)	(0.110)	(0.106)	(0.106)
Percent of bachelor’s degree	−166.6 *	−162.3 *	−47.71	−99.06
	(80.06)	(80.00)	(78.57)	(78.99)
BIPOC	−13,736 ***	−11,457 **	−5335	−12,329 ***
	(3506)	(3527)	(3450)	(3463)
Population aged 65 and above	−976.7	−1192	12,854	11,398
	(10,056)	(10,047)	(9867)	(9929)
Population log	−2828 ***	−2806 ***	−243.6	−1482 ***
	(421.6)	(421.3)	(424.4)	(420.3)
Bonus	−1665	−1466	−1937	38.91
	(1326)	(1325)	(1299)	(1353)
Lottery	2935 **	3236 ***	3003 **	−282.8
	(937.7)	(938.5)	(918.8)	(937.2)
Fear index × Per capita income		−0.00215 ***		
		(0.000386)		
Fear index × Unemployment rate			30.54 ***	
			(1.148)	
Bonus × Fear index				−80.64 ***
				(19.70)
Lottery × Fear index				143.1 ***
				(7.223)
Constant	24,028 ***	20,760 ***	−7491	8224
	(5131)	(5160)	(5165)	(5107)
Observations	16,976	16,976	16,976	16,976
Number of Counties	2856	2856	2856	2856

Note: This table reports the beta-coefficient. Standard errors in parentheses. *** *p* < 0.001, ** *p* < 0.01, * *p* < 0.05.

## Data Availability

Data supporting reported results are available publicly.
